# Non-centrosymmetric superconductor Th$_4$Be$_{{33}}$Pt$_{{16}}$ and heavy-fermion U$_4$Be$_{{33}}$Pt$_{{16}}$ cage compounds

**DOI:** 10.1038/s41598-021-01461-6

**Published:** 2021-11-16

**Authors:** P. Koželj, M. Juckel, A. Amon, Yu. Prots, A. Ormeci, U. Burkhardt, M. Brando, A. Leithe-Jasper, Yu. Grin, E. Svanidze

**Affiliations:** Max-Planck-Institut für Chemische Physik fester Stoffe, Nöthnitzer Straße 40, 01187 Dresden, Germany

**Keywords:** Materials chemistry, Physical chemistry, Chemical synthesis

## Abstract

Unconventional superconductivity in non-centrosymmetric superconductors has attracted a considerable amount of attention. While several lanthanide-based materials have been reported previously, the number of actinide-based systems remains small. In this work, we present the discovery of a novel cubic complex non-centrosymmetric superconductor ${\text {Th}}_4{\text {Be}}_{{33}}{\text {Pt}}_{{16}}$ ($I{\bar{4}}3d$ space group). This intermetallic cage compound displays superconductivity below $T_{\text {c}} = 0.90 \pm 0.04$ K, as evidenced by specific heat and resistivity data. ${\text {Th}}_4{\text {Be}}_{{33}}{\text {Pt}}_{{16}}$ is a type-II superconductor, which has an upper critical field ${\text {H}}_{{\text {c}}2} = 0.27$ T and a moderate Sommerfeld coefficient $\gamma _{\text {n}} = 16.3 \pm 0.8$ mJ ${\text {mol}}^{-1}_{\text {Th}}$ ${\text {K}}^{-2}$. A non-zero density of states at the Fermi level is evident from metallic behavior in the normal state, as well as from electronic band structure calculations. The isostructural ${\text {U}}_4{\text {Be}}_{{33}}{\text {Pt}}_{{16}}$ compound is a paramagnet with a moderately enhanced electronic mass, as indicated by the electronic specific heat coefficient $\gamma _{\text {n}} = 200$ mJ ${\text {mol}}^{-1}_{\text {U}}$ ${\text {K}}^{-2}$ and Kadowaki–Woods ratio $A/\gamma ^2 = 1.1 \times 10^{-5}$ $\upmu$ $\Omega$ cm ${\text {K}}^2$ ${\text {mol}}_{\text {U}}^2$ (mJ)$^{-2}$. Both ${\text {Th}}_4{\text {Be}}_{{33}}{\text {Pt}}_{{16}}$ and ${\text {U}}_4{\text {Be}}_{{33}}{\text {Pt}}_{{16}}$ are crystallographically complex, each hosting 212 atoms per unit cell.

## Introduction

The absence of inversion symmetry in non-centrosymmetric superconductors (NCSCs) allows an electronic asymmetric spin–orbit coupling (ASOC) to exist. This, in turn, may cause normally forbidden mixing of the spin-singlet and spin-triplet components into a new superconducting pairing state^1,2^. Broken inversion symmetry also opens up a possibility for unusual effects in areas of superconducting magnetic response, electromagnetic effects, superconducting finite moment states, as well as unusual surface states^1,2^. Among NCSCs, previous studies have concentrated on heavy-fermion materials such as ${\text {CePt}}_3$Si, ${\text {CeIrSi}}_3$^3–6^, and ${\text {CeRhSi}}_3$^7–12^, in which superconductivity coexists with antiferromagnetic order. However, in these materials it is impossible to disentangle the effects of non-centrosymmetricity from those of strong electron correlations. A possible solution to this issue is an in-depth investigation of weakly correlated NCSCs, such as ${\text {Li}}_2({\text {Pd}}_{1-x}{\text {Pt}}_x)_3$B^13–16^, ${\text {La}}_2{\text {C}}_3$^17–20^, and ${\text {Y}}_2{\text {C}}_3$^21–25^.

**Figure 1:**
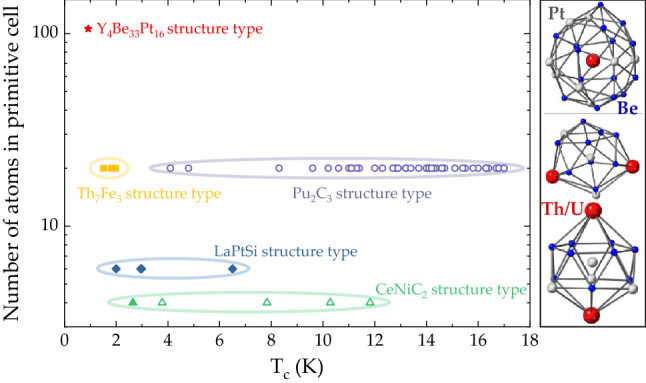
Crystallographic complexity and superconductivity in Th-based compounds. Left: The relation between the number of atoms per unit cell, i.e., structural complexity^26,27^, and the superconducting temperature $T_{\text {c}}$ for the three previously reported classes of Th-based NCSCs and for the newly discovered NCSC ${\text {Th}}_4{\text {Be}}_{{33}}{\text {Pt}}_{{16}}$ (structure type ${\text {Y}}_4{\text {Be}}_{{33}}{\text {Pt}}_{{16}}$). Right: Coordination polyhedra around the Th/U and Pt atoms in Th/${\text {U}}_4{\text {Be}}_{{33}}{\text {Pt}}_{{16}}$.

Interestingly, even though the presence of Th has been suggested to enhance ASOC^28,29^, only a handful of Th-based NCSCs are known. Crystallographically, it is possible to classify them into three groups according to the number of atoms per primitive unit cell, as shown in Fig. 1. The first group contains ${\text {ThCoC}}_2$-based superconductors (structure type ${\text {CeNiC}}_2$), namely stoichiometric ${\text {ThCoC}}_2$, and its substitutional derivates ${\text {ThCo}}_{{x}}{\text {Ni}}_{1-x}$C${_2}$ for $0.5\le x \le 0.9$^30,31^, La$_{1-x}$Th$_x$NiC${_2}$^32,33^, Y$_{1-x}$Th$_x$NiC${_2}$^34^. The second group includes superconductors with the LaPtSi structure type, namely Th*M*Si ($M=$ Ir, Co, Ni, Pt)^28,29,35–38^. The third group contains Th$_2$C$_3$^39–41^ (structure type Pu$_2$C$_3$) and the partially Th-substituted La and Y analogues, as well as superconductors with the Th$_{7}$M$_{3}$ (Th$_7$Fe$_{{3}}$ structure type ($M=$ Fe, Co, Ni, Ru, Rh, Os, and Ir)^42–46^ and the derived Th$_{{7}}$(Co,Fe)$_{3}$ pseudobinaries^47^. What all previously reported Th-based NCSCs have in common is their relative structural simplicity, as evidenced by the small number of atoms per primitive unit cell.

In this work, we present the discovery and characterization of a novel structurally complex NCSC Th$_4$Be$_{{33}}$Pt$_{{16}}$. This compound is isostructural to the previously reported family of intermetallic cage compounds $R_4$Be$_{{33}}$Pt$_{{16}}$ (*R* = Y, La–Nd, Sm–Lu), which host a wide range of physical properties^48,49^. The Th$_4$Be$_{{33}}$Pt$_{{16}}$ compound displays type-II superconductivity below $T_{\text {c}} = 0.90 \pm 0.04$ K, as inferred from the specific heat and electrical resistivity data. The upper critical field $H_{{\text {c}}2} = 0.27$ T is comparable to that of other conventional superconductors. In the normal state, Th$_4$Be$_{{33}}$Pt$_{{16}}$ displays metallic electrical resistivity and Sommerfeld coefficient $\gamma _{\text {n}} = 16.3 \pm 0.8$ mJ mol$^{-1}_{\text {Th}}$ K$^{-2}$, which are in good agreement with non-zero density of states at the Fermi level, illustrated by band structure calculation. With 212 atoms per unit cell, the Th$_4$Be$_{{33}}$Pt$_{{16}}$ compound is the most complex Th-based NCSC reported to date, see Fig. 1 for an overview. We also report synthesis and characterization of the isostructural U$_4$Be$_{{33}}$Pt$_{{16}}$ and (La$_{1-x}$Th$_{{x}}$)$_4$Be$_{{33}}$Pt$_{{16}}$ phases. No ordering has been observed in the U compound down to $T = 80$ mK. The specific heat data yield the electronic specific heat coefficient $\gamma _{\text {n}} = 200$ mJ mol$^{-1}_{\text {U}}$ ${\text {K}}^{-2}$, which classifies U$_4$Be$_{{33}}$Pt$_{{16}}$ as a moderately heavy heavy-fermion compound. The effective mass enhancement in the U$_4$Be$_{{33}}$Pt$_{{16}}$ compound is less than that, observed in unconventional superconductor UBe$_{{13}}$^50–52^—another heavy-fermion system containing Be and U. What both compounds have in common is a very strong coupling between minute changes in crystal chemistry and resultant physical properties^53^.

## Results

### Materials synthesis and characterization.

The results of the single crystal X-ray diffraction analysis of U$_4$Be$_{{33}}$Pt$_{{16}}$ and Th$_4$Be$_{{33}}$Pt$_{{16}}$ are summarized in Tables SI and SII of the “Supplementary Information”. As reported earlier for the isostructural $R_4$Be$_{{33}}$Pt$_{{16}}$ compounds^48^, they crystallize in the non-centrosymmetric space group $I{\bar{4}}3d$ and can be thought of as an arrangement of rod-like structures along the 3-fold [111] directions of the unit cell, consisting of interpenetrating coordination polyhedra centered on the Th and Pt1 atoms (see Fig. 1 (right)). The Th atoms are located at the center of a 20-atom polyhedra, consisting of Pt and Be (see Fig. 1 (right)). The Pt atoms have environment derived from Frank–Kasper polyhedrons and have coordination numbers of 14 (Pt1) and 13 (Pt2). With 212 atoms per unit cell, U$_4$Be$_{{33}}$Pt$_{{16}}$ and Th$_4$Be$_{{33}}$Pt$_{{16}}$ are classified as complex metallic alloys^27^.

**Figure 2:**
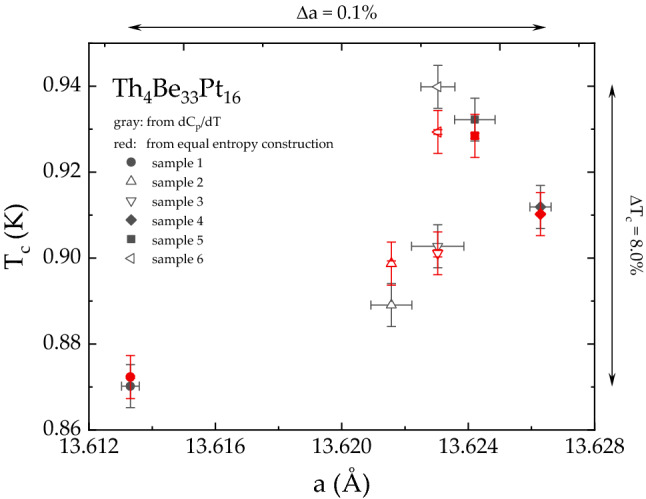
Relationship between crystallinity and superconducting properties of Th$_4$Be$_{33}$Pt$_{16}$. The critical temperature $T_{\text {c}}$ versus the lattice parameter *a* for six Th$_4$Be$_{{33}}$Pt$_{{16}}$ samples. The values of $T_{\text {c}}$ were taken from the equal entropy construction (red symbols) and as the minimum in the first derivative of the specific heat data (gray symbols). The variation of *a* is two orders of magnitude less, compared to the corresponding variation of $T_{\text {c}}$.

As is the case for $R_4$Be$_{{33}}$Pt$_{{16}}$^49^, both series of Th$_4$Be$_{{33}}$Pt$_{{16}}$ and U$_4$Be$_{{33}}$Pt$_{{16}}$ samples include minority secondary phases, see Figs.  S1–S4. The determined lattice parameters reveal clear differences between the samples, caused most probably by Be vacancies, like it was observed for Al in the UBe$_{13-x}$Al$_x$ material^53^. The possible vacancies in the Be sublattice are evident from the enhanced displacement of the Be1 and Be2 positions (Table SIIS). The exact homogeneity ranges of Th$_4$Be$_{33-x}$Pt$_{{16}}$ and U$_4$Be$_{33-x}$Pt$_{{16}}$ are difficult to describe quantitatively, due to the analytical problems in establishing the exact Be content. In particular, the superconducting temperature $T_{\text {c}}$ of the Th$_4$Be$_{{33}}$Pt$_{{16}}$ compound is strongly affected by the magnitude of the lattice parameter *a*. Although *a* varies by only $~ 0.1\%$ ($13.6133 \pm 0.0003$ Å  $\le a \le$ $13.6263 \pm 0.0003$ Å), $T_{\text {c}}$ changes over a range of $8\%$ (Fig. 2). For U$_4$Be$_{{33}}$Pt$_{{16}}$, the change in the lattice parameter *a* is also on the same order of magnitude $\Delta a = 0.1\%$ ($13.4907 \pm 0.0002$ Å  $\le a \le$ 13.5033 $\pm 0.0002$ Å).

### Superconductivity in Th$_4$Be$_{{33}}$Pt$_{{16}}$

**Figure 3:**
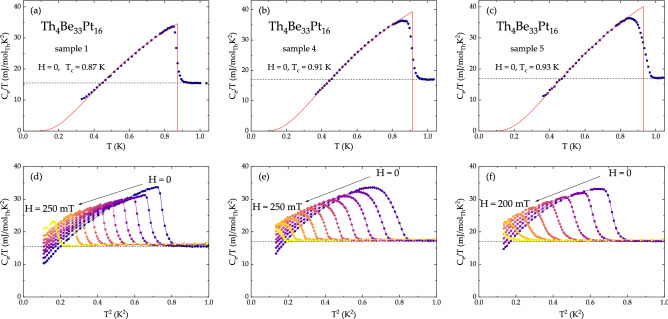
Superconducting properties of Th$_4$Be$_{33}$Pt$_{16}$. (**a**)–(**c**) The electronic specific heat of three Th$_4$Be$_{{33}}$Pt$_{{16}}$ samples, measured in $H = 0$. Note that a feature, associated with transition into superconducting state is observed around $T_{\text {c}} = 0.90 \pm 0.04$ K for all three samples. Vertical solid lines are equal entropy construction. The horizontal dashed line corresponds to the Sommerfeld coefficient $\gamma _n$. The red curve is a fit $C/T \propto {\text {e}}^{-\Delta /T}$. (**d**)–(**f**) Temperature-dependent specific heat data, scaled by temperature, as a function of temperature squared for three Th$_4$Be$_{{33}}$Pt$_{{16}}$ samples, measured in various magnetic fields.

A first indication of bulk superconductivity in Th$_4$Be$_{{33}}$Pt$_{{16}}$ is an anomaly, observed in the specific heat data (Fig. 3a–c and Fig. S6). A BCS-like transition occurs around $T_{\text {c}} = 0.90 \pm 0.04$ K, with its sharpness, height, and width varying slightly among the three samples. This variation can be attributed to the presence of impurity phases and an appreciable homogeneity range of Th$_4$Be$_{{33}}$Pt$_{{16}}$, as deduced from the powder X-ray diffraction data and energy dispersive micrographs, presented in Figs. S1 and S3, respectively. The two impurity phases, observed in Th$_4$Be$_{{33}}$Pt$_{{16}}$ samples are Be$_{{21}}$Pt$_5$ (superconductor, $T_{\text {c}} = 2.06$ K^54^) and ThPt (paramagnet^55^) in the amount of $< 1\%$ at. The physical properties of Th$_4$Be$_{{33}}$Pt$_{{16}}$ can therefore be decoupled from those of the impurity phases.

Upon subtraction of the phononic contribution to the specific heat ($\beta =1.44$ mJ mol$_{{\text {Th}}}^{-1}$ K$^{-4}$, $\gamma _{\text {n}} = 16.3 \pm 0.8$ mJ mol$^{-1}_{\text {Th}}$ K$^{-2}$), the electronic specific heat data, shown in Fig. 3a–c, are fit with $C/T \propto {\text {e}}^{-\Delta /T}$ (red line). Since the sharpness of the transition is sample-dependent, the value of $T_{\text {c}}$ was taken from the derivative of the specific heat data, shown in Fig. S6. The value of $\Delta C_{\text {e}}/T_{\text {c}} \gamma _{\text {n}} = 1.31 \pm 0.08$ is comparable to the BCS value of $\Delta C_{\text {e}}/T_{\text {c}} \gamma _{\text {n}} = 1.43$^56^. Using the McMillan formula, the magnitude of the Debye temperature $\theta _{\text {D}} = 262$ K yields a moderate value of the electron–phonon coupling $0.4< \lambda _{\text {e-p}} < 0.49$ (for $0.10 \le \mu ^* \le 0.15$)^57^. Upon application of magnetic field, the superconducting transition is gradually suppressed, as summarized in Fig. 3d–f. The corresponding values of $T_{\text {c}}$ are given in the $H -T$ phase diagram, shown Fig. 5a (circles). Based on the specific heat data, the behavior of Th$_4$Be$_{{33}}$Pt$_{{16}}$ is similar to that of a conventional, type-II superconductor. However, given that $\mu$SR (muon spin rotation, relaxation, and resonance) experiments can frequently reveal complex nature of NCSCs^58–61^, a future, in-depth $\mu$SR study of Th$_4$Be$_{{33}}$Pt$_{{16}}$, is likely to be very fruitful.

**Figure 4:**
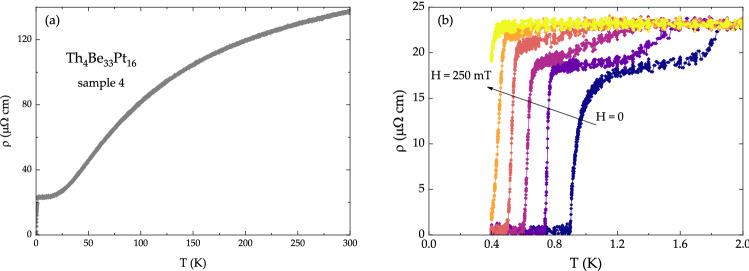
Electrical resistivity of Th$_4$Be$_{33}$Pt$_{16}$. (**a**) $H = 0$ and (**b**) $0 \le H \le 250$ mT data. High-temperature region indicates metallic behavior of Th$_4$Be$_{{33}}$Pt$_{{16}}$, while a drop around $T = 0.9$ K marks entrance into superconducting state. The additional transition around $T = 1.8$ K can be attributed to the secondary phase Be$_{{21}}$Pt$_5$.

**Figure 5:**
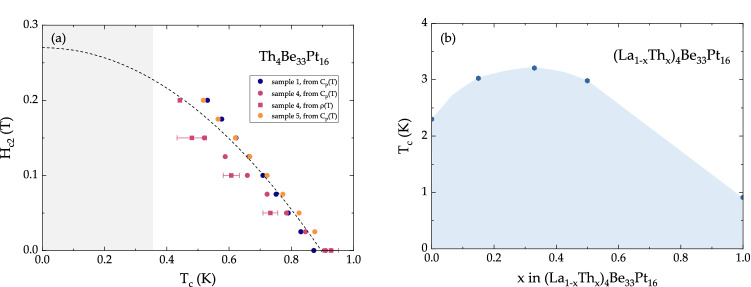
Evolution of superconducting properties in Th$_4$Be$_{33}$Pt$_{16}$ as a function of a tuning parameter. (**a**) The *H*–*T* phase diagram of Th$_4$Be$_{{33}}$Pt$_{{16}}$. The dashed black line corresponds to the Ginzburg–Landau fit of the data, while the red line is a linear fit around $T_{\text {c}}$ needed for the WHH extrapolation. (**b**) The value of the superconducting critical temperature $T_{\text {c}}$ as a function of *x* in (La$_{1-x}$Th$_{{x}}$)$_4$Be$_{{33}}$Pt$_{{16}}$.

The electrical resistivity of Th$_4$Be$_{{33}}$Pt$_{{16}}$, shown in Fig. 4a, classifies this material as a metal. At lower temperatures, a transition into superconducting state is observed around $T = 0.9$ K (Fig. 4b). A second, weaker transition, which occurs at $T = 1.8$ K, can be attributed to the Be$_{{21}}$Pt$_5$ superconductor ($T_{\text {c}} = 2.06$ K^54^). Upon application of magnetic field, both transitions shift down in temperature, allowing to extract $T_{\text {c}}$ values for Th$_4$Be$_{{33}}$Pt$_{{16}}$ in each respective magnetic field. The resultant values are summarized in Fig. 5a (squares). Given considerable overheating of the Th$_4$Be$_{{33}}$Pt$_{{16}}$ samples, the error bars for the values of $T_{\text {c}}$, extracted from the resistivity data are larger, than those for the $T_{\text {c}}$ values, extracted from the specific heat data. The magnetic susceptibility data, collected on all Th$_4$Be$_{{33}}$Pt$_{{16}}$ samples (not shown), classify this compound as a paramagnet with temperature-independent magnetic susceptibility between $T = 2$ K and $T = 300$ K. Pauli paramagnetic susceptibility in Th$_4$Be$_{{33}}$Pt$_{{16}}$ is consistent with metallic resistivity and non-zero density of states (DOS) at the Fermi level $E_{\text {F}}$, as evidenced by band structure calculations (see below).

Chemical substitution in Th-based NCSCs can sometimes lead to interesting results. For example, in unconventional NCSC ThCo$_{1-x}$Ni$_{{x}}$C$_{{2}}$ with $x = 0$–0.5, the superconducting transition temperature increases from 2.65 K at $x = 0$–12.1 K at $x = 0.4$^31^. A similar trend was also observed in (La$_{1-x}$Th$_{{x}}$)$_4$NiC$_{{2}}$ with $x = 0$–0.8, where $T_{\text {c}}$ varies from $\sim 2.80$ to 7.70 K, with the maximum $T_{\text {c}}$ observed at $x = 0.5$^32^. Given that we have previously reported superconductivity in La$_4$Be$_{{33}}$Pt$_{{16}}$ at $T_{\text {c}} = 2.5$ K^49^, we have investigated the (La$_{1-x}$Th$_{{x}}$)$_4$Be$_{{33}}$Pt$_{{16}}$ pseudo-ternary in hopes of increasing the value of $T_{\text {c}}$. As can be seen in Fig. 5b, the maximum value of $T_{\text {c}} = 3.21$ K is achieved for the $x = 0.33$ sample. We have therefore been able to achieve a $T_{\text {c}}$ enhancement of nearly $30\%$, compared to the $x = 0$ (La$_4$Be$_{{33}}$Pt$_{{16}}$) value or $250\%$, compared to the $x = 1$ (Th$_4$Be$_{{33}}$Pt$_{{16}}$) value. This indicates that future chemical substitution experiments among the isostructural rare-earth- and actinide-based $R/A_4$Be$_{{33}}$Pt$_{{16}}$ compounds are likely of interest, especially between superconducting and magnetic members of the series.

The values of $T_{\text {c}}$ in various fields for three Th$_4$Be$_{{33}}$Pt$_{{16}}$ samples are summarized in the *H*–*T* phase diagram (Fig. 5). The fit to the Ginzburg–Landau relation $H_{c2}(T)=H_{c2}(0) \left[ 1-(T/T_c)^2\right]$ yields $T_{\text {c}} = 0.90$ K and $H_{\text {c2}}(0) = 0.27$ T. An extrapolation using the Werthamer–Helfand–Hohenberg approximation^62,63^ $H_{c2}^{\text {WHH}}(0) = - 0.69\, T_c \left( dH_{c2}/dT_c\right) _{T = T_c}$ also gives similar values $T_c^{\text {WHH}} = 0.91$ K and $H_{c2}^{\text {WHH}}(0)= 0.30$ T. The magnitude of $H_{\text {c2}}$ is below the paramagnetic limit and is similar to that of other Th-based NCSCs–ThNiSi (0.058 or 0.126 T^28^), ThCoSi (4.5 T^29^), ThCoC$_{{2}}$ ($> 0.4$ T^30^), Th$_7$Co$_3$ ($\sim 1$ T^43^), and ThCo$_{{x}}$Ni$_{1-x}$C${_2}$ (1–10 T^31^).

**Figure 6:**
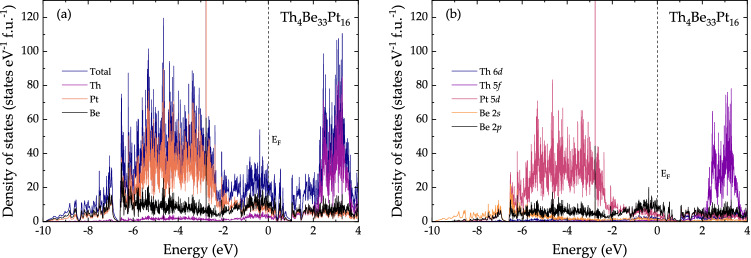
Computed electronic density of states at the fully-relativistic level for Th$_4$Be$_{{33}}$Pt$_{{16}}$: total and atom-resolved density of states (left), as well as projected density of states showing the contributions of the most relevant orbitals (right).

The spin un-polarized scalar relativistic calculations place the Fermi level near a local minimum of the DOS with $N(E_{\text {F}}) = 17.05$ states eV$^{-1}$ f.u.$^{-1}$. In contrast, at the same level of calculation the Fermi level was found near a local maximum in Y$_4$Be$_{{33}}$Pt$_{{16}}$^48^. The spin polarized calculations at both scalar and fully-relativistic levels converged to zero spin magnetic moment solution—also for all individual atoms. The orbital moments, too, were zero in the fully relativistic calculation. The total, atom-, and atomic orbital-resolved DOS, computed at the fully relativistic level for Th$_4$Be$_{{33}}$Pt$_{{16}}$, are shown in Fig. 6. The value of $N(E_{\text {F}})$ is 19.92 states eV$^{-1}$ f.u.$^{-1}$, yielding $\gamma _{\text {theory}} = 11.7$ mJ mol$^{-1}_{\text {Th}}$  $\text {K}^{-2}$), which is similar to $\gamma _{\text {n}}$, extracted from the specific heat data. Similarly to the case of Y$_4$Be$_{{33}}$Pt$_{{16}}$, three energy regions can be recognized in the occupied part. The lower-energy region (below $-6.58$ eV) is dominated by Be 2*s* and Pt 6*s* contributions with Be 2*p* and Pt 5*d* states becoming also important above $-7.6$ eV. The middle-energy region (between $-6.58$ and $-2.20$ eV) consists mainly of Pt 5*d* contributions. The upper-energy region, dominated by Be 2*p* states, contains contributions from Pt 5*d* and Th 6*d* as well as Th 5*f*, Pt 6*s* and Be 2*s*. The unoccupied part features two pseudo gaps at 0.40 and 1.04 eV, the latter originating from a band (Fig. S5) very similar to the one observed in Y$_4$Be$_{{33}}$Pt$_{{16}}$^48^. The Th 5*f* states behave band-like with an occupancy of about 0.45 electrons, therefore, there is no need for a correlated system treatment. The splitting of the 6*p* semi-core energies due to the spin–orbit coupling is about 7.36 eV. The $6p_{1/2}$ states are located between $-22.75$ and $-22.72$ eV with a narrow band width of 0.03 eV, while the $6p_{3/2}$ states have a wider spread of 0.29 eV (between $-15.52$ and $-15.23$  eV). The weighted average of these values is $-17.83$ eV, which is very close to the energy of the 6*p* states in the scalar relativistic calculation, $-17.90$ eV.

### Heavy-fermion behavior in U$_4$Be$_{{33}}$Pt$_{{16}}$

**Figure 7:**
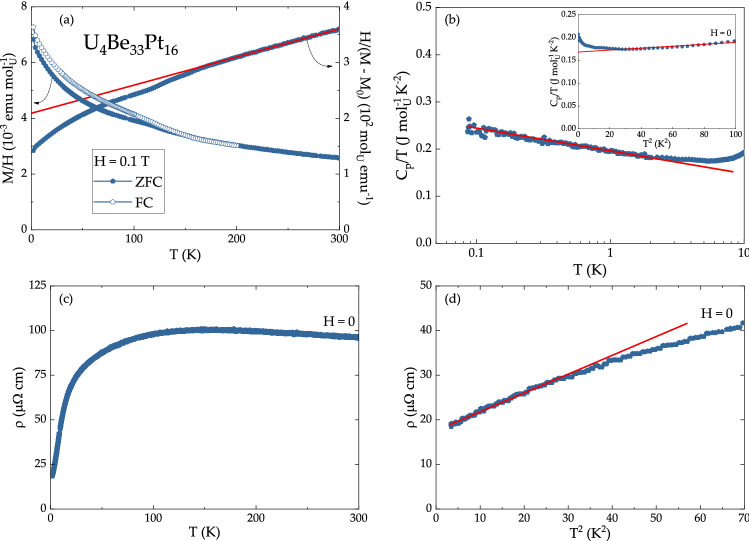
(**a**) Zero-field-cooled and field-cooled magnetic susceptibility (left axis) and inverse magnetic susceptibility (right axis) data for U$_4$Be$_{{33}}$Pt$_{{16}}$ in $H = 0.1$ T. The solid line is a Curie–Weiss fit to the inverse susceptibility. (**b**) Low-temperature specific heat data exhibit logarithmic divergence over more than a decade in temperature (red line). Inset: $C_{\text {p}}/T$ vs. $T^2$ with the solid line representing the fit from which the value of $\gamma _{\text {n}}$ was extracted. (**c**) Temperature-dependent resistivity data for U$_4$Be$_{{33}}$Pt$_{{16}}$ in $H = 0$. (**d**) The low-temperature region of the $\rho$ vs. $T^2$ plot.

The physical properties of the isostructural U$_4$Be$_{{33}}$Pt$_{{16}}$ compound were also investigated. The magnetic susceptibility data, shown in Fig. 7a indicate paramagnetic behavior for the temperature range between $T = 2$ K and $T = 300$ K. The zero-field-cooled (ZFC) (full symbols) and field-cooled (FC) (open symbols) data show a small bifurcation around $T = 120$ K. The origin of the ZFC/FC splitting is a small ferromagnetic impurity, present in elemental uranium. The linear fit to the inverse magnetic susceptibility data above $T = 150$ K (right axis, straight line) yields Weiss temperature $\Theta _{\text {W}} = - 135$ K and effective moment $\mu _{\text {eff}} = 2.39 \mu _{\text {B}}/{\text {U}}$. The value of $\mu _{\text {eff}}$ is smaller than that expected for U$^{4+}$ and U$^{3+}$ configurations ($3.58\mu _{\text {B}}/{\text {U}}$ and $3.62\mu _{\text {B}}/{\text {U}}$, respectively), suggesting possible delocalization of 5*f* orbitals and their hybridization with conduction electrons. The value of $M/H (T = 1.8~{\text {K}}, H = 0.1~{\text {T}}) = 7 \times 10^{-3}$ emu mol$_{\text {U}}^{-1}$ is rather small compared to that of compounds, located close to a ferromagnetic quantum critical point—for example, UNiAl ($22 \times 10^{-3}$ emu mol$_{\text {U}}^{-1}$^64^) and UCoAl ($55 \times 10^{-3}$ emu mol$_{\text {U}}^{-1}$^65^). Instead, the value of *M*/*H* in U$_4$Be$_{{33}}$Pt$_{{16}}$ is similar to that of UAu$_2$ ($7.5 \times 10^{-3}$ emu mol$_{\text {U}}^{-1}$^66^).

While no ordering has been observed down to $T = 80$ mK, the low-temperature specific heat data show logarithmic divergence over a decade in temperature—see Fig. 7b. The specific heat data are fit to the $C_{\text {p}}/T = \gamma + \beta T -T/T_0 (logT)$ function, yielding an enhanced value of the electronic specific heat coefficient $\gamma _{\text {n}} = 200$ mJ mol${^{-1}}_{\text {U}}$ K$^{-2}$. The possibility of the electron mass enhancement arising from the small atomic percentage of U atoms can be eliminated by considering that while $\gamma _{\text {n}}$ of isostructural Th$_4$Be$_{{33}}$Pt$_{{16}}$ is only $16.3 \pm 0.8$ mJ mol$^{-1}_{\text {Th}}$ K$^{-2}$, the corresponding value for U$_4$Be$_{{33}}$Pt$_{{16}}$ is larger by more than one order of magnitude. Therefore, U$_4$Be$_{{33}}$Pt$_{{16}}$ is classified as a moderately heavy heavy-fermion system.

The electrical resistivity of U$_4$Be$_{{33}}$Pt$_{{16}}$ exhibits a drop around $T = 100$ K, marking the onset of Kondo scattering (Fig. 7c). The residual resistivity ratio (RRR) of 5.1 is similar to what is typically seen in polycrystalline samples and larger than that observed in systems with disorder^67^. In Fig. 7d, the resistivity is plotted as a function of $T^2$, allowing to extract the value of coefficient $A = 0.43~{\upmu } \Omega$ cm K$^{-2}$. The resultant Kadowaki–Woods ratio $A/\gamma ^2 = 1.1 \times 10^{-5}~{\upmu } \Omega$ cm K$^2$ mol$_{\text {U}}^2$ (mJ)$^{-2}$ supports correlated electron behavior in U$_4$Be$_{{33}}$Pt$_{{16}}$^68–70^.

## Discussion and conclusions

In this work we present two isostructural compounds—Th$_4$Be$_{{33}}$Pt$_{{16}}$ and U$_4$Be$_{{33}}$Pt$_{{16}}$—that show drastically different properties. The Th$_4$Be$_{{33}}$Pt$_{{16}}$ compound is a NCSC with a transition temperature of $T_c = 0.90 \pm 0.04$ K and upper critical field $H_{{\text {c}}2}(0) = 0.27$ T. The bulk superconductivity in Th$_4$Be$_{{33}}$Pt$_{{16}}$ is confirmed by measurements of specific heat and resistivity, which identify it as a weakly-coupled BCS-like superconductor. Metallic behavior above the superconducting transition is consistent with non-zero density of states at the Fermi level, as inferred from electronic band structure calculations. Negligible mass enhancement in Th$_4$Be$_{{33}}$Pt$_{{16}}$ is evident from the electronic specific heat coefficient $\gamma _{\text {n}} = 16.3 \pm 0.8$ mJ  mol$^{-1}_{\text {Th}}$ K$^{-2}$. The U$_4$Be$_{{33}}$Pt$_{{16}}$ compound, on the other hand, shows a large $\gamma _{\text {n}} = 200$ mJ mol$^{-1}_{\text {U}}$ K$^{-2}$ and Kadowaki–Woods ratio $A/\gamma ^2 = 1.1 \times 10^{-5}~{\upmu } \Omega$ cm K$^2$ mol$_{\text {U}}^2$ (mJ)$^{-2}$, indicating heavy-fermion behavior in this system.

## Methods

All sample preparation was performed in the specialized laboratory, equipped with an argon-filled glove box system (MBraun, $p(H_2O/O_2) < 0.1$ ppm)^71^. Polycrystalline samples of U$_4$Be$_{{33}}$Pt$_{{16}}$ and Th$_4$Be$_{{33}}$Pt$_{{16}}$ were prepared by arc-melting U (wires, Good Fellow, $>99.9 \%$) or Th (foil, Good Fellow, $>99.9 \%$) with Be (sheet, Heraeus, $>99.9 \%$) and Pt (balls, Chempur, $>99.9 \%$) with the compositions, shown in Table 1. The melting temperature of the Th$_4$Be$_{{33}}$Pt$_{{16}}$ compound is $T_{\text {m}} = 1289$ ${^{\circ }}$C, while that of U$_4$Be$_{{33}}$Pt$_{{16}}$ is $T_{\text {m}} = 1350$ ${^{\circ }}$C. Therefore, all samples were annealed for 4 days at $T = 1115$ ${^{\circ }}$C. Small inclusions of secondary phases were identified by several experimental methods, see Figs. S1–S4. Based on these data, only three out of six Th$_4$Be$_{{33}}$Pt$_{{16}}$ samples (samples 1, 4, and 5) and one out of six U$_4$Be$_{{33}}$Pt$_{{16}}$ (sample 3), which show the least amount of impurities, were characterized in-depth in the present study. Since the amount of Be is hard to quantify analytically (see below), and given that the evaporation losses of Be are unavoidable, the only way to keep control of the sample composition is to follow a careful weighing protocol. None of the samples exhibited any marked air or moisture sensitivity.

**Table 1:** Sample summary for U$_4$Be$_{{33}}$Pt$_{{16}}$ and Th$_4$Be$_{{33}}$Pt$_{{16}}$ samples.

Sample number	Th$_4$Be$_{{33}}$Pt$_{{16}}$		U$_4$Be$_{{33}}$Pt$_{{16}}$	
	Nominal composition	Resultant composition^a^	Nominal composition	Resultant composition^a^
1	${\text {Th}}_{9.8}{\text {Be}}_{60.8}{\text {Pt}}_{29.4}$	${\text {Th}}_{10.0}{\text {Be}}_{59.9}{\text {Pt}}_{30.1}$	${\text {U}}_{8.0}{\text {Be}}_{67.8}{\text {Pt}}_{24.2}$	${\text {U}}_{10.0}{\text {Be}}_{59.9}{\text {Pt}}_{30.1}$
4	${\text {Th}}_{6.0}{\text {Be}}_{70.1}{\text {Pt}}_{23.9}$	${\text {Th}}_{6.9}{\text {Be}}_{65.4}{\text {Pt}}_{27.7}$	${\text {U}}_{6.3}{\text {Be}}_{68.6}{\text {Pt}}_{25.1}$	${\text {U}}_{7.4}{\text {Be}}_{63.1}{\text {Pt}}_{29.5}$
2	${\text {Th}}_{6.4}{\text {Be}}_{67.8}{\text {Pt}}_{25.8}$	${\text {Th}}_{7.3}{\text {Be}}_{63.4}{\text {Pt}}_{29.3}$	${\text {U}}_{6.1}{\text {Be}}_{69.4}{\text {Pt}}_{24.5}$	${\text {U}}_{7.1}{\text {Be}}_{64.4}{\text {Pt}}_{28.5}$
3	${\text {Th}}_{6.2}{\text {Be}}_{68.8}{\text {Pt}}_{25.0}$	${\text {Th}}_{7.1}{\text {Be}}_{64.4}{\text {Pt}}_{28.5}$	${\text {U}}_{6.0}{\text {Be}}_{70.3}{\text {Pt}}_{23.7}$	${\text {U}}_{6.8}{\text {Be}}_{65.9}{\text {Pt}}_{27.3}$
6	${\text {Th}}_{5.6}{\text {Be}}_{72.1}{\text {Pt}}_{22.3}$	${\text {Th}}_{6.5}{\text {Be}}_{67.4}{\text {Pt}}_{26.1}$	${\text {U}}_{5.8}{\text {Be}}_{71.1}{\text {Pt}}_{23.1}$	${\text {U}}_{6.7}{\text {Be}}_{66.4}{\text {Pt}}_{26.9}$
5	${\text {Th}}_{5.8}{\text {Be}}_{71.2}{\text {Pt}}_{23.0}$	${\text {Th}}_{6.7}{\text {Be}}_{66.3}{\text {Pt}}_{27.0}$	${\text {U}}_{5.6}{\text {Be}}_{72.0}{\text {Pt}}_{22.4}$	${\text {U}}_{6.6}{\text {Be}}_{67.2}{\text {Pt}}_{26.2}$

^a^Estimated assuming that the mass, lost during arc-melting, is solely that of Be.

Powder X-ray diffraction was performed on a Huber G670 Image plate Guinier camera with a Ge-monochromator (CuK$\alpha _1$, $\lambda$ = 1.54056 Å). Phase identification was done using the WinXPow software^72^. The data for all Th$_4$Be$_{{33}}$Pt$_{{16}}$ and U$_4$Be$_{{33}}$Pt$_{{16}}$ samples are shown in Figs. S1 and S2, respectively. The lattice parameters were determined by a least-squares refinement using the peak positions, extracted by profile fitting (WinCSD software^73^). Single crystal diffraction data were collected using a Rigaku AFC7 diffractometer, equipped with a Saturn 724+ CCD detector and a MoK$\alpha$ radiation source ($\lambda$ = 0.71073 Å). The SHELXL software was used for data analysis. The results of the crystallographic characterization are provided in Tables SI and SII.

Chemical composition of polished samples was studied using energy-dispersive X-ray spectroscopy with a Jeol JSM 6610 scanning electron microscope equipped with an UltraDry EDS detector (ThermoFisher NSS7). The semi-quantitative analysis was performed with 25 keV acceleration voltage and $\approx 3$ nA beam current. Small inclusions of secondary phases are also visible from back-scatter scanning electron micrographs, presented in Figs. S3 and S4. However, it has to be emphasized that the Be content cannot be reliably analyzed by this method.

The magnetic properties were studied using a Quantum Design (QD) Magnetic Property Measurement System for the temperature range from $T = 1.8$ K to $T = 300$ K and for applied magnetic fields up to $H = 7$ T. The inverse magnetic susceptibility data were fit to the Curie–Weiss law, after a temperature-independent contribution $M_0 = 2 \times 10^{-4}$ emu mol$_{\text {U}}^{-1}$ has been subtracted. The specific heat data were collected on a QD Physical Property Measurement System (PPMS) in the temperature range from $T = 0.4$ K to $T = 10$ K for magnetic fields up to $H = 9$ T. The *dc* resistivity measurements in a temperature range from $T = 1.8$ K to $T = 300$ K were carried out using the standard four-probe method in the QD PPMS. Platinum wires were attached to the polished surfaces of bar-shaped sample using silver epoxy.

First-principles electronic structure calculations were carried out by using the all-electron full-potential local orbital (FPLO) method^74^. Exchange-correlation effects were taken into account by the local density approximation to the density functional theory as parametrized by Perdew and Wang^75^. Both scalar and fully-relativistic treatments were employed. The implementation of the latter in the FPLO method is based directly on the Dirac equation^76,77^. Brillouin zone integrations were evaluated using a $6 \times 6 \times 6$ *k*-mesh and the linear tetrahedron method.

## Supplementary information

Supplementary Information.
