# Supplementary material for: Non-centrosymmetric superconductor Th4Be33Pt16 and heavy-fermion U4Be33Pt16 cage compounds
Source: Sci Rep. 2021 Nov 16;11:22352. doi: 10.1038/s41598-021-01461-6 (PMC8595440; doi:10.1038/s41598-021-01461-6)
Supplement: Supplementary file 1 — Supplementary Information. [file 41598_2021_1461_MOESM1_ESM.pdf]

Supplementary information

Supplementary Table I. Crystallographic data for $U_4Be_{33}Pt_{16}$ and $Th_4Be_{33}Pt_{16}$ (structure type $Y_4Be_{33}Pt_{16}$).

Composition	$U_4Be_{33}Pt_{16}$	$Th_4Be_{33}Pt_{16}$
Space group	$I\bar{4}3d$ (Nr. 220)	
Pearson symbol	$cI212$	
Formula units per unit cell	$Z = 4$	
Lattice parameters		
a (Å)	13.4990(4)	13.6367(4)
V (Å^3)	2459.8(2)	2535.9(2)
Calculated density (g cm^{-3})	11.80	11.38
Crystal form	prism-like	irregular shaped
Crystal size (μm)	$25 \times 40 \times 60$	$35 \times 40 \times 50$
Diffraction system	RIGAKU AFC7	
Detector	Saturn 724+ CCD	
Radiation type, λ (Å)	MoKα_1, 0.71073	
Scan; step (°); N (images)	ϕ, 0.6, 600	ϕ, 0.6, 1200
Maximal 2 θ (°)	63.9	84.0
	$-18 \le h \le 19$	$-11 \le h \le 25$
Range in h, k, l	$-18 \le k \le 19$	$-25 \le k \le 12$
	$-19 \le l \le 8$	$-25 \le l \le 13$
Absorption correction	multi-scan	
T(max)/T(min)	5.15	2.94
Absorption coefficient (mm^{-1})	120.4	114.8
$N(hkl)$ measured	8148	17978
$N(hkl)$ unique	710	1479
R_{int}	0.072	0.099
$N(hkl)$ observed	705	1475
Observation criteria	$F(hkl) \ge 4\sigma(F)$	
Refined parameters	25	27
Flack parameters	0.000(5)	0.06(3)
R_F	0.029	0.044
R_W	0.031	0.046
Residual peaks / (e Å^{-3})	-0.97/1.27	-2.29/2.91

Supplementary Table II. Atomic coordinates and equivalent (isotropic) displacement parameters (in Å^2) in the crystal structures of $U_4Be_{33}Pt_{16}$ and $Th_4Be_{33}Pt_{16}$.

Atom	Site	x/a	y/b	z/c	U_{eq}[a]/U_{iso}[b]
		$U_4Be_{33}Pt_{16}$			
U1	16c	0.8079(5)	x	x	0.0064(1)[a]
Be1	12a	5/8	0	3/4	0.007
Be2	24d	0.917(3)	0	3/4	0.007
Be3	48e	0.057(2)	0.987(2)	0.665(2)	0.007
Be4	48e	0.622(2)	0.918(2)	0.892(2)	0.007
Pt1	16c	0.93576(5)	x	x	0.0088(2)[a]
Pt2	48e	0.48789(5)	0.89827(5)	0.77454(5)	0.0064(2)[a]
		$Th_4Be_{33}Pt_{16}$			
Th	16c	0.80789(4)	x	x	0.00811(8)[a]
Be1	12b	5/8	0	3/4	0.033(15)
Be2	24d	0.910(4)	0	3/4	0.027(8)
Be3	48e	0.059(2)	0.988(2)	0.664(2)	0.010(3)
Be4	48e	0.622(2)	0.915(2)	0.893(2)	0.014(4)
Pt1	16c	0.93509(4)	x	x	0.00877(8)[a]
Pt2	48e	0.48956(4)	0.89746(4)	0.77612(4)	0.0083(1)[a]

[a] $U_{eq} = \frac{1}{3}[U_{11} \cdot a^{*2} \cdot a^2 + ... + 2 \cdot U_{23} \cdot b^* \cdot c^* \cdot b \cdot c \cdot cos(a)]$
[b] $U_{iso}(Be1) = U_{iso}(Be2) = U_{iso}(Be3) = U_{iso}(Be4)$ constrained for $U_4Be_{33}Pt_{16}$

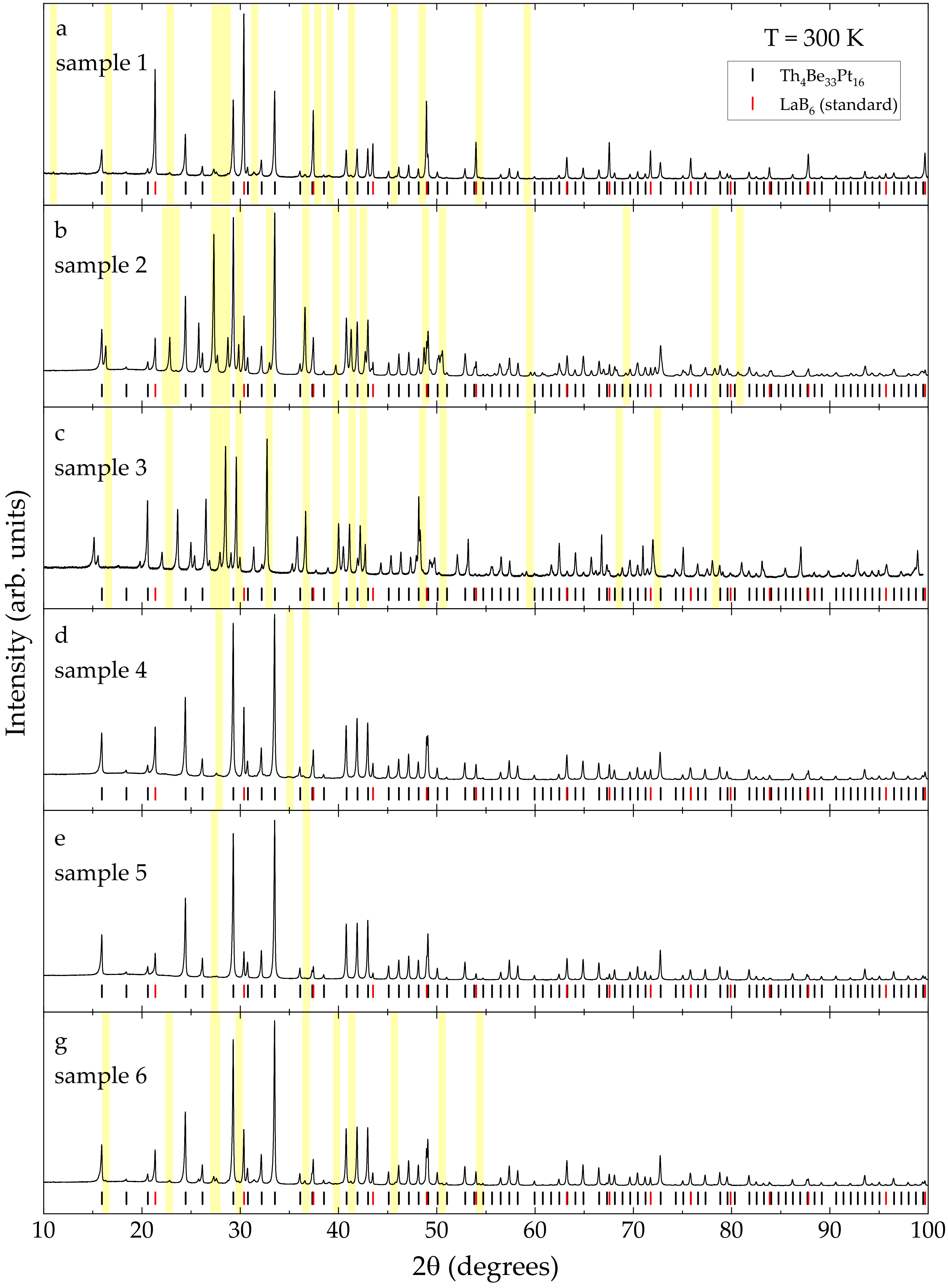

Supplementary Figure 1. Powder x-ray diffraction patterns (Cu$K\alpha_1$) for six $Th_4Be_{33}Pt_{16}$ samples, with their compositions

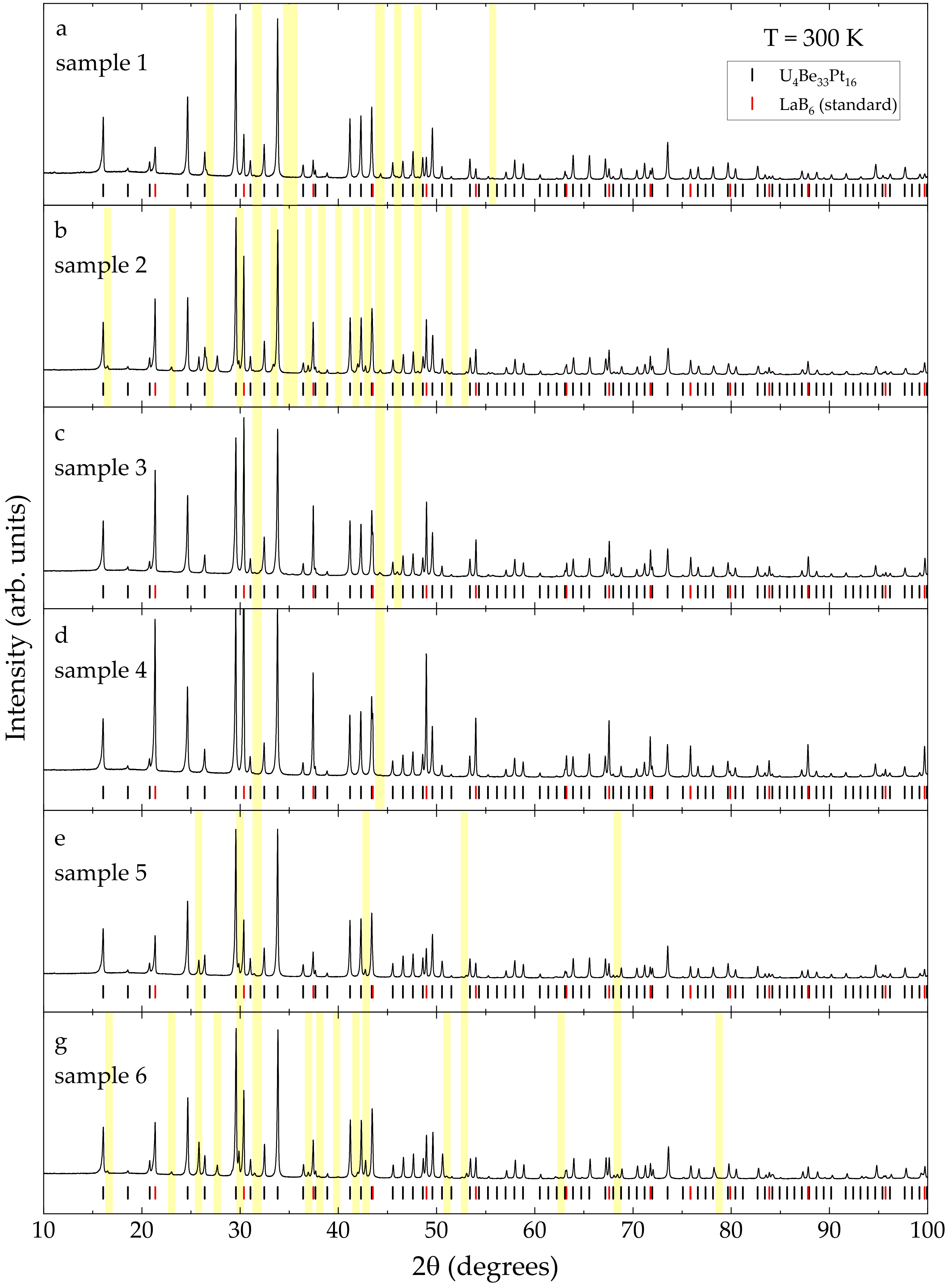

Supplementary Figure 2. Powder x-ray diffraction patterns (Cu$K\alpha_1$) for six $U_4Be_{33}Pt_{16}$ samples, with their compositions listed

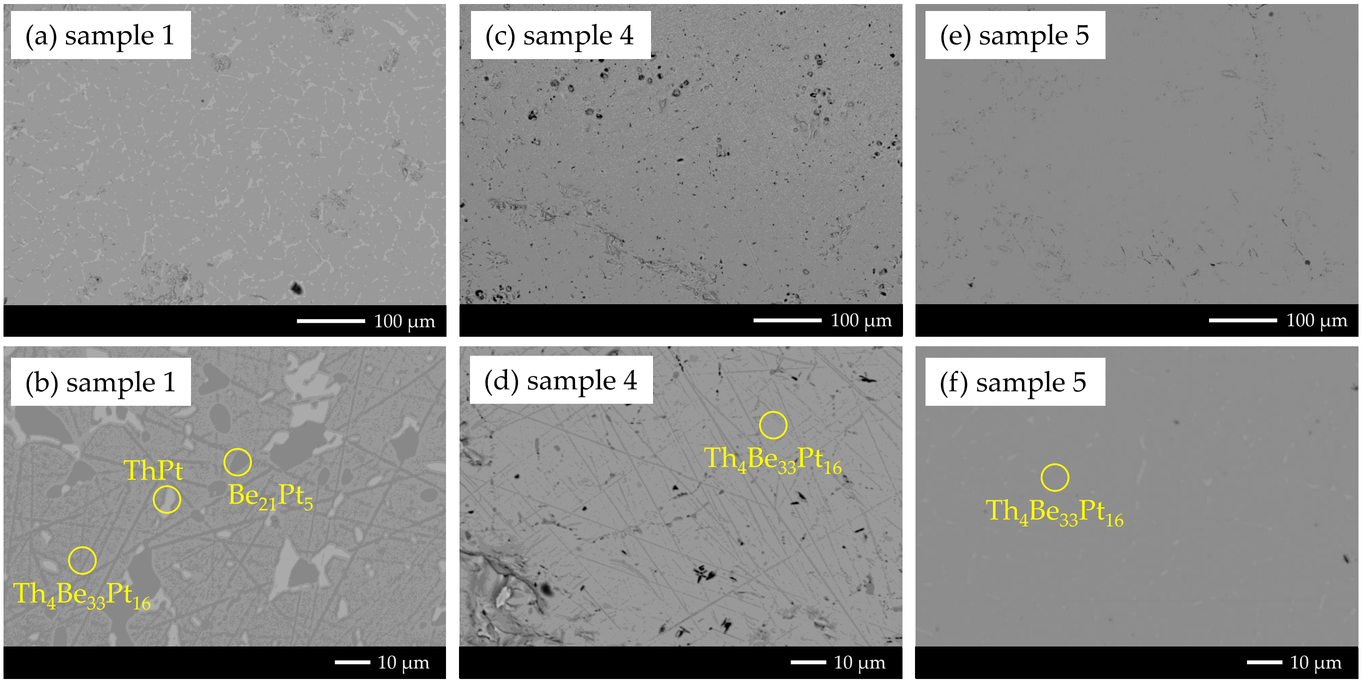

Supplementary Figure 3. Back-scatter scanning electron micrographs (U_{acc} = 25 kV) for $Th_4Be_{33}Pt_{16}$ samples, with their compositions listed in Table I. The majority $Th_4Be_{33}Pt_{16}$ phase corresponds to light gray regions, while the impurity phases $Be_{21}Pt_5$ and ThPt correspond to dark gray and white regions respectively.

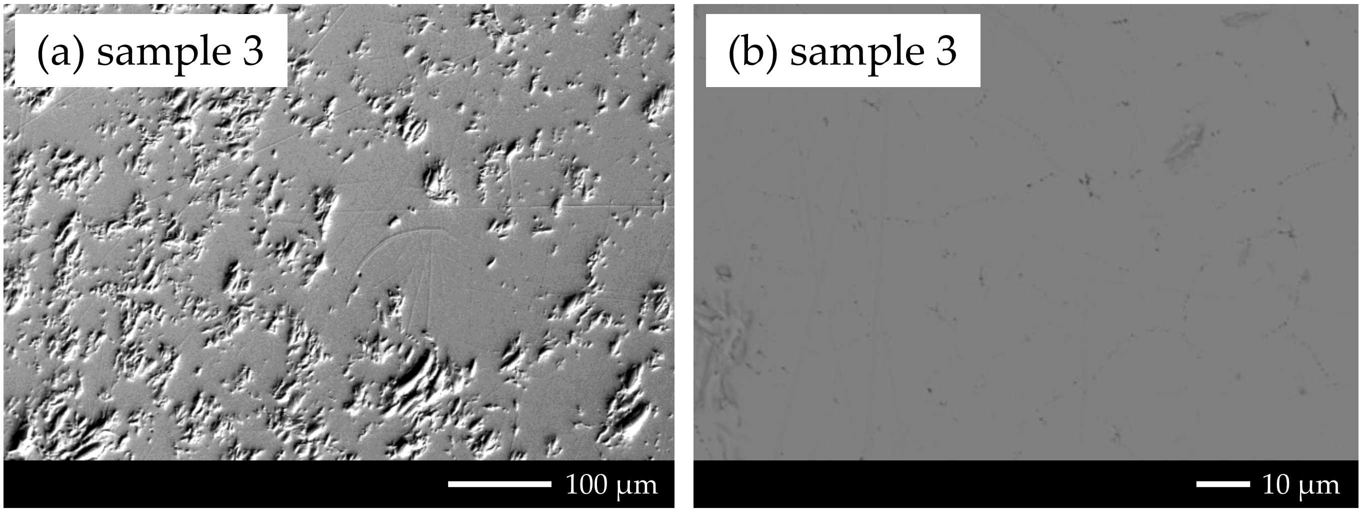

Supplementary Figure 4. (a) Secondary electron image and (b) back-scatter electron micrographs (U_{acc} = 25 kV) for $U_4Be_{33}Pt_{16}$ sample, with their compositions listed in Table I. The majority $U_4Be_{33}Pt_{16}$ phase corresponds to light gray regions, while the impurity phases $Be_{21}Pt_5$ and ThPt correspond to dark gray and white regions respectively.

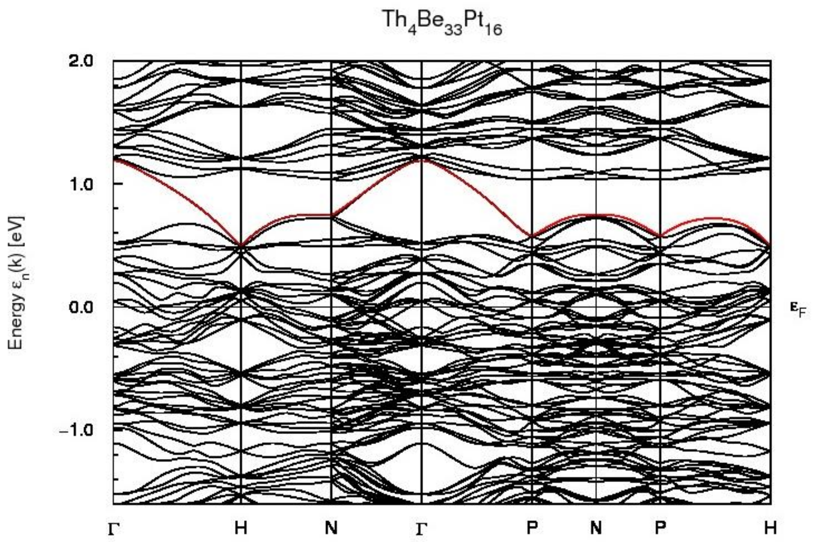

Supplementary Figure 5. Computed electron energy bands at the fully-relativistic level for $Th_4Be_{33}Pt_{16}$. The single band dispersing between 1.19 eV (at Γ) and 0.5 eV (at H) is responsible for the pseudo gap at 1.04 eV (shown by red solid line).

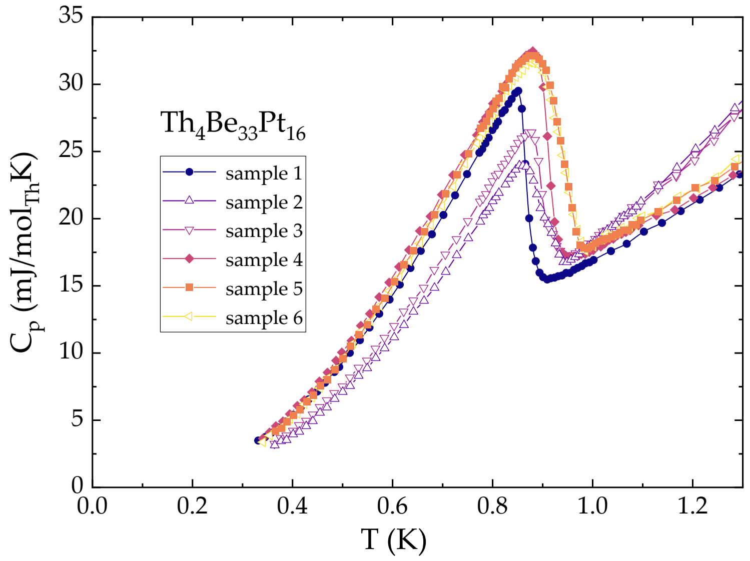

Supplementary Figure 6. Specific heat as a function of temperature for all six $Th_4Be_{33}Pt_{16}$ samples, measured in $H = 0$. Minor variations in the width and height of the feature can be attributed to the crystal chemistry of the $Th_4Be_{33}Pt_{16}$ phase. Specific heat data, taken in various magnetic fields for samples 1, 4, and 5, can be found in Fig. 3(a), (b), and (c), respectively.
